# Transcriptome Profile Analysis of Sugarcane Responses to *Sporisorium scitaminea* Infection Using Solexa Sequencing Technology

**DOI:** 10.1155/2013/298920

**Published:** 2013-10-23

**Authors:** Qibin Wu, Liping Xu, Jinlong Guo, Yachun Su, Youxiong Que

**Affiliations:** Key Lab of Sugarcane Biology and Genetic Breeding, Ministry of Agriculture, Fujian Agriculture and Forestry University, Fuzhou, Fujian 350002, China

## Abstract

To understand the molecular basis of sugarcane-smut interaction, it is important to identify sugarcane genes that respond to the pathogen attack. High-throughput tag-sequencing (tag-seq) analysis by Solexa technology was performed on sugarcane infected with *Sporisorium scitaminea*, which should have massively increased the amount of data available for transcriptome profile analysis. After mapping to sugarcane EST databases in NCBI, we obtained 2015 differentially expressed genes, of which 1125 were upregulated and 890 downregulated by infection. Gene ontology (GO) analysis revealed that the differentially expressed genes involve in many cellular processes. Pathway analysis revealed that metabolic pathways and ribosome function are significantly affected, where upregulation of expression dominates over downregulation. Differential expression of three candidate genes involved in MAP kinase signaling pathway, *ScBAK1* (GenBank Accession number: KC857629), *ScMapkk* (GenBank Accession number: KC857627), and *ScGloI* (GenBank Accession number: KC857628), was confirmed by reverse transcription polymerase chain reaction (RT-PCR). Real-time quantitative PCR (qRT-PCR) analysis concluded that the expression of these genes were all up-regulated after the infection of *S. scitaminea* and may play a role in pathogen response in sugarcane. The present study provides insights into the molecular mechanism of sugarcane defense to *S. scitaminea* infection, leading to a more comprehensive understanding of sugarcane-smut interaction.

## 1. Introduction

Sugarcane is the most important sugar crop and accounts for more than 90 percent of total sugar production in China [1]. Sugarcane smut, caused by *Sporisorium scitaminea*, is an important sugarcane fungal disease worldwide and is also one of the most important sugarcane diseases in China [2]. It was firstly reported in 1887 from Natal, South Africa [1]. The loss of stalk yield for smut susceptible varieties is serious [3, 4]. Nowadays, sugarcane smut is prevalent in sugarcane producing areas all over the world and has caused the elimination of several sugarcane varieties with high yield and high sugar [5]. Because of this, smut resistance is a major objective of sugarcane variety breeding [6, 7].

Cultivation of resistant varieties is the most effective disease-control measure [6, 8]. In order to understand the sugarcane smut defense mechanism, research has been conducted on sugarcane bud morphology, cytology, and physiology with smut resistance [8–10]. Padmanaban et al. [11] studied the relationship between bud structure and the smut resistance of sugarcane varieties. The results revealed that the presence of resistance was associated with certain bud morphologies. Identification of differentially expressed genes under various stresses can give clues as to defence mechanisms and biochemical pathways regulated under each stress [12]. At present, research has focused on the molecular interaction between plant and pathogen using various techniques, including microarrays [13], representational difference analysis (RDA) [14], suppression subtractive hybridization (SSH) [15], cDNA-amplified fragment length polymorphism (cDNA-AFLP) [16], and serial analysis of gene expression (SAGE) [17]. As for the molecular mechanism of sugarcane-smut interaction, there have been several previous reports [18–24]. In the research of Thokoane and Rutherford [18], a cDNA-AFLP technique was applied to detect differential gene expression in smut resistant and susceptible sugarcane genotypes. Sequence homology analysis indicated that a Pto ser/threo protein kinase and an active gypsy-type LTR retro-transposon played a role in the response of sugarcane to the infection of *S. scitaminea*. Heinze et al. [19] conducted another study using suppression subtractive hybridization (SSH) method, which revealed sugarcane genes encoding proteins homologous to chitinases, as well as transcripts related to flavonoids, were involved in the sugarcane resistance after 7 days of *S. scitamineum* infection. Borrás-Hidalgo et al. [20] obtained 62 differentially expressed genes before and after the infection of *S. scitaminea*, among which 52 were upregulated and 10 were downregulated. LaO et al. [21] investigated differential expression by cDNA-AFLP method in the susceptible and the resistant genotypes. A total of 64 genes were proved to be differentially expressed, among which 67.2% were upregulated in the resistant cultivar. This result indicated that sugarcane response involved genes of the oxidative burst, defense response, and ethylene and auxin pathways. Que et al. [22] obtained 7 differentially expressed genes before and after inoculation of cultivars NCo376 and F134 with smut by DDRT-PCR. In Que et al. [23], 136 transcript-derived fragments (TDFs) were found to be differentially expressed in response to challenge by *S. scitaminea*. Que et al. [24] applied 2-DE and MALDI-TOF-TOF/MS to reveal the protein expression profile of sugarcane after inoculating with *S. scitamineum*. This is the first report of proteomic investigation of sugarcane exposed to* S. scitamineum*.

As discussed above, the mechanism of sugarcane smut defense has been studied using various techniques. However, sugarcane is a highly heterozygous crop and there is no genomic resource for this genus, so more attention is needed to establish the molecular interactions between sugarcane and pathogen. Differentially expressed genes can be investigated using a multitude of methods, such as cDNA microarray, cDNA-AFLP, RDA, SAGE, and SSH [13–17]. However, these techniques have some inherent limitations, such as an inability to detect low expression levels, and cross-hybridization problems [25–27]. The Solexa sequencing technology enables many applications, including whole genome resequencing, transcriptome sequencing, gene expression profiling, and epigenomic sequencing. The technique uses sequencing-by-synthesis on an eight-channel flow-cell to produce more than 10 million reads per channel with read lengths up to 100 bp [27–34]. Digital gene expression profiling (DGE) in the Illumina Solexa sequencing platform is capable of simultaneously sequencing a large number of DNA molecules and has become a powerful tool to detect the changes in gene expression [25, 28]. DGE can yield millions of short reads (32–40 nt) and is more suitable for tag-based transcriptome sequencing [25, 28].

The application of Solexa technology for the identification of differentially expressed genes associated with the molecular mechanism of interaction between the sugarcane-smut was therefore explored. In the present study, the Illumina Solexa sequencing technology was used to reveal the response of sugarcane to infection by *S. scitaminea*. Yacheng 05–179, a highly resistant sugarcane genotype, was used as plant material. RNA expression profile sequencing analysis of two samples, before and after sugarcane smut fungus inoculation, was then conducted by Solexa sequencing. This study aims to discover the pathogenesis-related differentially expressed genes and further understand the interaction mechanisms between sugarcane and *S. scitaminea* at the molecular level.

## 2. Materials and Methods

### 2.1. Plant Materials and Treatment

The sugarcane genotypes, Yacheng 05–179 and Liucheng 03–182, were chosen as smut-resistant and smut-susceptible plant material, respectively. These two sugarcane genotypes were provided by the Key Lab of Sugarcane Biology and Genetic Breeding, Ministry of Agriculture (Fuzhou, China). Sugarcane smut fungus (*S. scitaminea*) was collected from Liucheng 03–182 plants and stored at 4°C.

A smut spore suspension of 5 × 10^6^ spores/mL was needle-inoculated on to sugarcane buds as the treatment group, while sterile water inoculated buds were the mock group [9, 10]. These sugarcane buds were then cultured in an incubator at 28°C ± 0.5°C, and phenotypically normal bud tissue was then collected at time points of 12, 24, 36, 48, and 72 hours, respectively. All samples were immediately frozen in liquid nitrogen and stored in a refrigerator at −80°C until RNA extraction.

### 2.2. RNA Isolation

Total RNA was isolated using the TRIzol reagent according to the manufacturer's instructions (Invitrogen). Dried RNA samples were dissolved in diethylpyrocarbonate-treated H_2_O, and RNA quality was assessed on 1.0% denaturing agarose gels. RNA quality and quantity were verified using a NanoDrop 1000 spectrophotometer and an Agilent 2100 Bioanalyzer prior to Solexa sequencing at BGI. Total RNA samples of Yacheng 05–179 before and after pathogen inoculation at the time point of 48 h were subjected to digitized expression profiling sequencing at Beijing Genomics Institute (BGI; Shenzhen, China). Both RNA samples of Yacheng 05–179 and Liucheng 03–182 were used for Real-time quantitative PCR at time point 12, 24, 36, 48, and 72 hours.

### 2.3. Solexa Sequencing

The gene expression libraries were prepared using Illumina Gene Expression Sample Prep Kit according to the manufacturer's instructions. Specified Experimental Process, use magnetic oligo (dT) beads adsorption to purify mRNA from 6 *μ*g total RNA, and then the first and second strand cDNA was synthesized. The bead-bound cDNA was subsequently digested with restriction enzyme *Nla* III, which recognized and cut off the CATG sites. The 3′ cDNA fragments attached to the oligo (dT) beads were washed away, and then 5′ cDNA fragments were ligated to the Illumina Adaptor 1, which contained a recognition site for the endonuclease *Mme* I for cutting 17 bp downstream of the recognition site (CATG), producing tags with adaptor 1. After removing 3′ fragments with magnetic beads precipitation, Illumina adaptor 2 is ligated to the 3′ ends of tags, acquiring tags with different adaptors of both ends to form a tag library. After 15 cycles of linear PCR amplification, 95 bp fragments are purified by 6% TBE PAGE Gel electrophoresis. The two constructed tag libraries were fixed onto the Illumina Sequencing Chip (flowcell) for cluster generation through situ amplification and were deep-sequenced using Illumina Genome Analyzer. After image analysis, base calling, and quality calibration, the raw data was produced [25, 26].

### 2.4. RT-PCR Confirmation and qRT-PCR Analysis of Three Candidate Genes

The expressions of three candidate genes in MAP kinase signaling pathway were determined by RT-PCR and Real-time quantitative PCR (qRT-PCR). Gene-specific primers were designed according to the gene sequences using the Primer Premier 5.0 software and were synthesized commercially (Shanghai Sangon, China).

The first-strand cDNA was synthesized from total RNA using PrimeScript 1st strand cDNA synthesis kit (Takara). The primers for RT-PCR confirmation are listed in Table 1. The 25 *μ*L reaction mix contained 2.5 *μ*L 10 × PCR Buffer (plus Mg^2+^), 2.5 *μ*L deoxynucleotide triphosphates (dNTPs) (2.5 mM), 1.0 *μ*L first-strand cDNA, 1.0 *μ*L each of forward and reverse primers (10 *μ*M), and 0.125 *μ*L Ex-Taq enzyme (5 U/*μ*L). The ddH_2_O was added as supplement. The PCR amplification program consisted of predenaturation for 4 min at 94°C; denaturation for 1 min at 94°C, annealing for 1 min at annealing temperature, and extension for 1.5 min at 72°C for 35 cycles; and a final extension for 10 min at 72°C. Then, 5 *μ*L of the product was electrophoresed on 1.0% agarose gel and viewed under UV light.

The *25S rRNA* (BQ536525) gene was chosen as the internal control in the qRT-PCR analysis [35]. The first-strand cDNA for qRT-PCR was synthesized using PrimeScript RT reagent kit (Takara). The primers for qRT-PCR analysis are listed in Table 2. In qRT-PCR analysis, 20 *μ*L samples were run on the ABI PRISM7500 real time PCR System using SYBR Green PCR Master Mix (Applied Biosystems). The qRT-PCR reaction conditions were held at 50°C for 2 min, predenatured at 95°C for 10 min, and then kept at 94°C for 15 s and at 60°C for 60 s for 40 cycles. Three replicas were set for each sample. When the reaction was completed and the melting curve was analyzed. The 2^−△△CT^ method was adopted to analyze the qRT-PCR results [36].

## 3. Results and Analysis

### 3.1. Solexa Sequencing of Infected Sugarcane

After filtering 3′ adapter sequence, empty reads, low-complexity reads, and low-quality reads, a total of 4,847,568 and 4,883,691 21 bp length clean tags were obtained that corresponded to 446,284 and 423,464 distinct tags for Yacheng 05–179 before and after *S. scitaminea* inoculation, respectively (Table 3).

Matching the tags to genes is an important step to annotate sequences and can reveal the molecular events behind the gene expression [26]. In this study, all clean tags were aligned to the reference sugarcane EST database in NCBI (http://www.ncbi.nlm.nih.gov/). After comparing with sugarcane EST database, 61.36% and 57.88% of clean tags, and 21.43% and 18.81% of distinct clean tags could be matched exactly with the reference sequences in two samples, respectively. Then, there are 639,019 and 610,306 clean tags for inoculation and mock libraries are uniquely mapped to database, while the number of distinct clean tags are 72812 and 64,815 (Table 3; see Supplementary Material available online at http://dx.doi.org/10.1155/2013/298920), respectively. However, because of incomplete sequences, there are a large proportion total clean tags (about 30%) and distinct tags (about 65%) which could not be aligned to the reference sequences (Supplementary Material).

**Figure 1:**
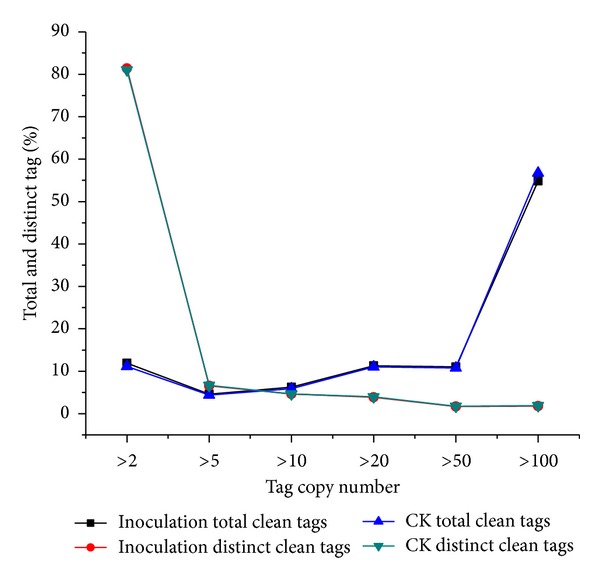
Distribution of total clean tag and distinct clean tag counts.

Heterogeneity and redundancy are two significant characteristics of mRNA expression [26]. We analyzed the distribution of clean tag copy number in the two libraries and found that the copy number of total clean tags and distinct clean tags showed very similar tendencies for two samples, respectively (Supplementary Material). In regard to the detection of low abundance expressed genes, the proportion of tags greater than 100-fold was over 54% and the proportion of distinct clean tags which was greater than 100-fold was very small (1.82% and 1.92%), but there are more than 80% distinct clean tags with a ratio within 5-fold (Figure 1 and Supplementary Material).

### 3.2. Differentially Expressed Genes in Two Libraries

By using blast against the reference sugarcane EST database in NCBI and putative differentially expressed genes were selected based on the following two criteria: (1) if the average fold change of gene expression before and after *S. scitaminea* inoculation was more than or equal to twofold (|log⁡_2_^Ratio^| ≥ 1) and (2) if the false discovery rate value of the single sample was less than 0.001 (FDR ≤ 0.001) [25]. By using this approach we obtained 2015 differentially expressed genes, and the expression of 1125 genes were identified as upregulated in the Yacheng 05–179 after inoculation as compared with that in Yacheng 05–179 before inoculation (Supplementary Material). The expression of 890 genes was decreased by more than twofold in Yacheng 05–179 before inoculation. We then screened 48 candidate genes for further study (31 upexpressed and 17 downexpressed) using the conditions of FDR ≤ 0.001 and |log⁡_2_^Ratio^| ≥ 5, and 3 upexpressed genes in the MAP kinase signaling pathway (Table 4).

### 3.3. Gene Annotation and Functional Classification

**Figure 2:**
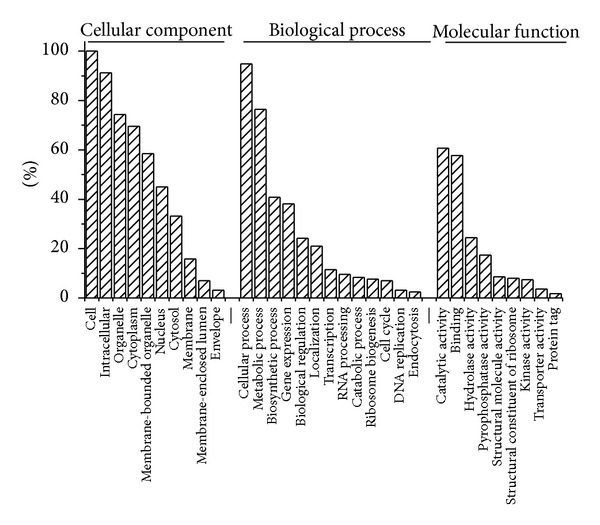
Gene ontology analysis for differentially expressed genes obtained using Solexa sequencing.

**Figure 3:**
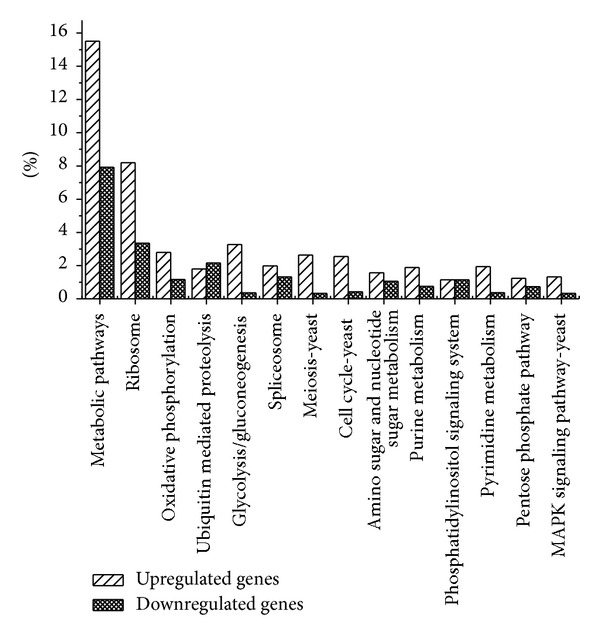
Pathway classifications for upregulated and downregulated genes.

**Figure 4:**
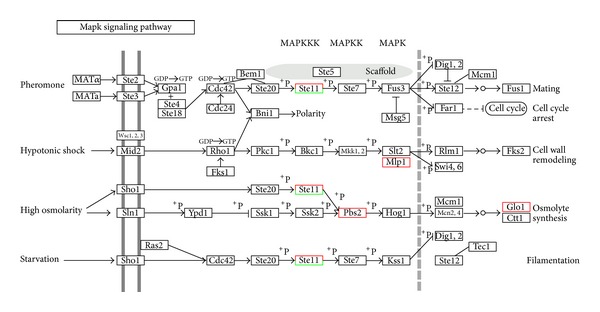
MAP kinase signaling pathway of sugarcane infected by *S. scitaminea*.

Gene ontology (GO) analysis was performed by mapping each differentially expressed gene on to the reference sugarcane EST database in NCBI (http://www.ncbi.nlm.nih.gov/). As shown in Figure 2, the proportions and comparisons of these differentially expressed genes were summarized in three main functional categories, cellular component with ten categories of genes, biological process with thirteen categories of genes, and molecular function with nine categories of genes. What should be stressed is that among these ten types of genes involved in molecular function, catalytic activity and binding are major molecular function (Figure 2 and Supplementary Material). This suggests that biological metabolism and catalytic activity are the major responsive classes; biological metabolic processes are enhanced and catalytic activity increases after the inoculation with the pathogen. In addition, the products of different genes usually cooperate with each other to exercise their biological function, so pathway-based analysis helps to further understand genes biological functions. We obtained 303 differentially expressed genes with pathway annotation involving in 79 biological pathways (Supplementary Material), among which metabolic pathways and ribosome were major pathways, and the number of upexpressed genes was significantly higher than that of those downexpressed (Figure 3). As indicated in Figure 4, it is interesting that, when sugarcane was infected by smut pathogen, the expression of three key enzymes, *ScBAK*1, *ScMapkk*, and *ScGloI*, which belongs to MAP kinase signaling pathway, were upregulated (Figure 4).

### 3.4. RT-PCR Confirmation and qRT-PCR Analysis of Three Candidate Genes

**Figure 5:**
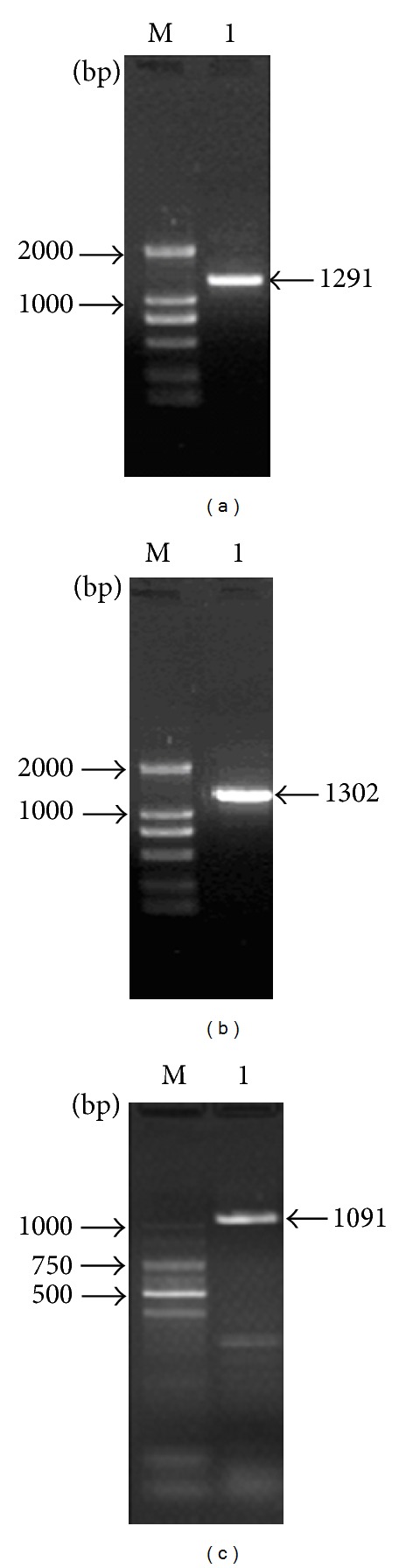
RT-PCR products for amplification of three upregulated genes in MAP kinase signaling pathway. (a) M, DL2000; Lane 1, PCR product of *ScBAK1* gene; (b) M, DL2000; Lane 1, PCR product of *ScMapkk* gene; (c) M, 100 bp Ladder; Lane 1, PCR product of *ScGloI* gene.

Three differentially expressed genes screened in Solexa sequencing were confirmed by RT-PCR. Full-length cDNA sequence of three upregulated genes in MAP kinase signaling pathway designated as *ScBAK1* (GenBank Accession number: KC857629), *ScMapkk* (GenBank Accession number: KC857627), and *ScGloI* (GenBank Accession number: KC857628) were obtained from sugarcane based on the bud full-length cDNA library, of which the length is 1,291 bp, 1,302 bp, and 1,091 bp, with ORF length of 1,004 bp, 1,068 bp, and 885 bp, respectively (Figure 5).

**Figure 6:**
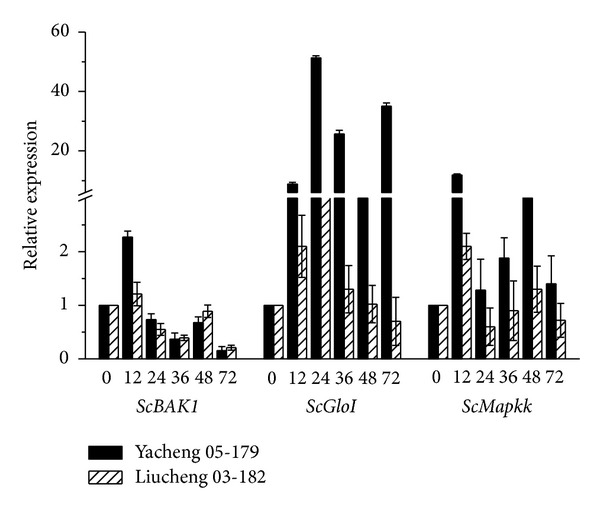
qRT-PCR expression profiles for *ScBAK1*, *ScGloI,* and *ScMapkk* gene under *S. scitaminea* stress in resistant and susceptible sugarcane genotypes.

qRT-PCR analysis was applied to validate the expressions of *ScBAK1*, *ScMapkk,* and *ScGloI* genes during the infection with *S. scitaminea* (Figure 6 and Supplementary Material). The smut-resistant genotype Yacheng 05–179 and smut-susceptible genotype LiuCheng 03–182 were selected as plant material. The results showed that during 0 h–12 h after pathogen infection, the expression of these three genes in both smut-resistant genotype and smut-susceptible genotype were up-regulated, but their expressions in smut-resistant genotype were much more significant than that in the smut-susceptible genotype especially for *ScMapkk* and *ScGloI* genes. Therefore, the expression of these genes may play a role in defence during *S. scitaminea* infection. However, the expression of *ScGloI* gene was up-regulated again after infection for 48 h in both genotypes of the resistant and susceptible one, and the expression of *ScMapkk* gene was up-regulated significantly again after 72 hpi in resistant Yacheng 05–179 but not in susceptible LiuCheng 03–182. This suggests that the smut-resistance function mechanism is different between these three genes, *ScBAK1*, *ScMapkk,* and *ScGloI*.

## 4. Discussion

The transcriptome, which can vary with external environmental conditions, is the set of all RNA molecules, including mRNA, rRNA, tRNA, and noncoding RNAs produced in one or a population of cells [32]. Transcriptome analysis using high-throughput short-read sequencing technology, such as Solexa sequencing, is straightforward, and does not have to be restricted to the genome of model organisms [25, 28, 29]. This analysis can provide information on gene expression and gene regulation and thus is essential to interpret the functional elements of the genome and reveal molecular mechanisms [32]. The nonmodel species that lack a reference genome, where RNA-Seq analysis has been applied, include *Eucalyptus grandis* [29], *Persea americana* [30], *Artemisia annua* [31], *Vitis vinifera* L. [28], *Sesamum indicum* L. [32], *Cicer arietinum* [34], *Medicago sativa* L. [33], and *Myrica rubra* [27].

Solexa sequencing is a high-throughput, short-read, massively parallel sequencing platform, of which the read length is relatively short (21 bp), and bioinformatics analysis of the corresponding differentially expressed genes has to only rely on sugarcane EST databases. Therefore, in the absence of corresponding sequenced genome information as a reference, many of the differentially expressed genes cannot be functionally annotated. However, the genomes of sorghum, maize and rice can act as reference information. According to sugarcane UniGenes identified and annotated by RNA-Seq and sorghum, maize and rice reference genome, we hope to establish a platform for future genetic and functional genomic research in sugarcane. In the present study, a total of 4,847,568 and 4,883,691 21 bp length clean tags that corresponded to 446,284 and 423,464 distinct tags for Yacheng 05–179 before and after *S. scitaminea* inoculation were successfully obtained. The results showed that Solexa technology can quickly assess millions of short sequences of both high-abundance and low-abundance expressed genes in sugarcane, similar to previous research reports [27–34]. This study was restricted by the absence of sugarcane genome sequence, thus, yielded an incomplete picture, and the completion of the sugarcane genome project should significantly contribute to a more detailed picture of the gene expression profiles related to sugarcane-smut interaction mechanism.

Differential gene expression during sugarcane-smut interaction is likely to be induced following pathogen challenge, which leads to up or downregulation of gene expression [18, 20, 22–24]. The influence of environment, evolution of *S. scitaminea* and molecular changes of the host sugarcane variety makes the interactions between sugarcane, the environment, and *S. scitaminea* more complicated. Previous studies on sugarcane-smut interaction revealed that sugarcane genes encoding proteins homologous to chitinases as well as transcripts related to the pathways of both phenylpropanoids and flavonoids were shown to be involved in the sugarcane resistance after 7 days of *S. scitamineum* infection [19]. Sugarcane-smut interaction was also carried out using a somaclonal genotype showing stable resistance for longer than 10 years [20]. Two months after fungal inoculation, some differentially expressed TDFs were similar to those identified in this study, that is, genes encoding NBS-LRR-like proteins, protein kinases, and proteins related to both auxin and ethylene pathways. In our study, we used samples after sugarcane smut fungus inoculation to characterize the transcriptome using Solexa sequencing. This generated more than 4.8 million tags of two samples after sequencing. However, there are a vast majority of tags (Table 3 and Supplementary Material, about 30% of total clean tags and about 65% of distinct tags) which cannot be aligned to the reference sequences. The functional categorization shows a complex linkage between genes involved in cellular and metabolic processes, gene expression, biological regulation, and localization. As indicated in Figure 3, metabolic pathway and ribosome were major pathways. Theoretically, the identification of differentially expressed genes has great potential to help to understand the molecular mechanism of smut resistance.

The study provides the potential to develop a molecular marker for smut resistance selection if the up-regulation of expression of *ScMapkk* and *ScGloI* genes could be verified in further smut-resistant and smut-susceptible genotypes with different genetic backgrounds. This is important for sugarcane smut resistance breeding because the interaction of sugarcane, smut pathogen and environment leads to the instability of the resistance phenotype tested by field-inoculation. In addition, only 48 candidate genes were screened in this study, and there are still many differentially expressed genes to be tested.

Plants possess integrated signaling networks that mediate the perception and responses to biotic and abiotic stresses which govern plant growth and development. MAP kinase signaling pathway is a conserved signaling pathway common in plants. The present results permit us to hypothesize about the role of the MAP kinase pathway on the sugarcane early response against the *S. scitaminea* infection. When subjected to biotic and abiotic stress, the MAP kinase signaling pathway is activated [37–40] and plays an important role in plant resistance mechanisms [41, 42]. The results of qRT-PCR analysis revealed that the expression of these three candidate genes, *ScBAK1*, *ScMapkk*, and *ScGloI* were up-regulated in both smut-resistant genotype and smut-susceptible genotype, but the expression in smut-resistant genotype was greater than that in the smut-susceptible genotype especially for *ScMapkk* and *ScGloI* genes. The expression of these three genes may play a role in defending against infection by pathogens in sugarcane. When sugarcane was infected by the smut pathogen, the expression of key enzymes were up-regulated, leading to the activation of MAP kinase signaling pathway and the triggering of the genes involved with plant defence (Figure 4), which was similar to the previous reports [37–40].

## 5. Conclusions

In conclusion, the usefulness of the Solexa sequencing in identifying genes related to sugarcane smut defense has been successfully demonstrated in this study. However, most of the molecular mechanisms of sugarcane-smut interaction are as yet unknown. More genes related to sugarcane defense and their expression profiles in response to smut infection should be analyzed further. The present study provides a Solexa sequencing platform for gene expression research on this crop and also a reference for studying the molecular mechanism in nonmodel organisms.

## Supplementary Material

Additional File 1: Statistics of clean tag alignment.pdf
Additional File 2: Distribution of clean tag copy number.pdf
Additional File 3: 2015 differentially expressed genes identified in sugarcane.xls
Additional File 4: Gene ontology analysis for differentially expressed genes.xls
Additional File 5: Pathway annotation of 305 differently expressed genes in sugarcane.xls
Additional File 6: Raw data of qRT-PCR for ScBAK1, ScMapkk and ScGloI genes.xls

## Figures and Tables

**Table 1:** Primers used for RT-PCR analysis.

Genes	Forward primer (5′-3′)	Reverse primer (5′-3′)	Product size (bp)
*ScBAK1 *	TTTGAGTGGTCCAATCCC	CGAGTCATCCGTCAGGTC	1,291
*ScMAPKK *	CCTTCTTGGGTTCTTCCTCC	ATCCCTTCTCATAGTCTCATCTAG	1,302
*ScGloI *	AGCCAGAAGAAAGGGAGC	GTTCATCAAGGCGGAAAC	1,091

**Table 2:** Primers used for qRT-PCR analysis.

Genes	Forward primer (5′-3′)	Reverse primer (5′-3′)	Product size (bp)
*25S rRNA *	GCAGCCAAGCGTTCATAGC	CCTATTGGTGGGTGAACAATCC	109
*ScBAK1 *	ACCTATGCCAATGTCTTACGG	GATGAAGCCAGTTGTAGCACC	168
*ScMAPKK *	TGAACTGCGGCTCAATCAAAG	TGCCTCACTAGCTGGACAACA	180
*ScGloI *	TGGACCGCACAATCAAATACTACAC	CAAAGCCCGTTCCAATGTCATAC	108

**Table 3:** Categorization and abundance of clean tags.

Summary	Inoculation	Control
Clean tags		
Total number	4,847,568	4,883,691
Distinct tag number	446,284	423,464
All tag mapping to genes		
Total number	2,974,532	2,826,151
Total % of clean tag	61.36%	57.88%
Distinct number	95,633	82,429
Distinct tag % of clean tag	21.43%	18.81%
One tag mapping to unique genes		
Total number	639,019	610,306
Distinct number	72,812	64,815
One tag mapping to multiple genes		
Total number	1,996,930	1,902,489
Distinct number	85,308	75,512
Unknown tags		
Total number	1,376,922	1,532,237
Total % of clean tag	28.40%	31.37%
Distinct number	288,186	283,137
Distinct tag % of clean tag	64.57%	66.86%

**Table 4:** Some selected differentially expressed genes identified using Solexa sequencing in sugarcane.

Gene	log⁡_2_^Ratio^	*P* Value	FDR	Annotation
gi|35266134	10.6	4.44*E* ^−16^	2.90*E* ^−14^	*Zea mays *STIP1 and U box protein 1
gi|35111854	9.2	1.67*E* ^−09^	4.70*E* ^−08^	*Sorghum bicolor *hypothetical protein
gi|34977253	9.0	1.35*E* ^−08^	3.42*E* ^−07^	*Saccharum* SP-80-3280 chloroplast
gi|35208604	8.8	1.09*E* ^−07^	2.43*E* ^−06^	*S. bicolor *hypothetical protein
gi|35293529	8.8	1.09*E* ^−07^	2.43*E* ^−06^	*S. bicolor *hypothetical protein
gi|26879857	8.8	2.19*E* ^−07^	4.62*E* ^−06^	*Mus musculus* chromosome 1
gi|34921564	8.6	8.82*E* ^−07^	1.69*E* ^−05^	*S. bicolor *hypothetical protein
gi|34942866	8.6	8.82*E* ^−07^	1.69*E* ^−05^	*S. bicolor* ATP synthase complex subunit 9
gi|34967006	8.5	3.55*E* ^−06^	5.99*E* ^−05^	*Z. ays *transcription factor GT-3b
gi|36001135	8.5	3.55*E* ^−06^	5.99*E* ^−05^	*Z. mays* omega-3 fatty acid desaturase
gi|35094785	8.4	7.14*E* ^−06^	0.000113	*S. bicolor* hypothetical protein
gi|35229762	8.4	7.14*E* ^−06^	0.000113	*Z. mays* nucleotide adenylyltransferase 1
gi|35294230	8.4	7.14*E* ^−06^	0.000113	*Z. mays* clone 294529 ATEB1A mRNA
gi|35981936	8.3	1.43*E* ^−05^	0.000214	*Z. mays* dihydrolipoamide S-acetyltransferase 1
gi|34965850	8.3	1.43*E* ^−05^	0.000214	*Z. mays* h/ACA ribonucleoprotein mRNA
gi|34942070	8.3	1.43*E* ^−05^	0.000214	*S. bicolor* mitochondrion
gi|35052194	8.3	1.43*E* ^−05^	0.000214	*S. officinarum* receptor kinase 1 (BAK1)
gi|35275212	8.3	1.43*E* ^−05^	0.000214	*S. bicolor* hypothetical protein
gi|34917382	8.2	2.88*E* ^−05^	0.000401	*S. bicolor *hypothetical protein
gi|34973370	8.2	2.88*E* ^−05^	0.000401	*Z. mays* myb-like protein mRNA
gi|34942843	8.2	2.88*E* ^−05^	0.000402	*S. bicolor* hypothetical protein
gi|35074510	8.1	5.77*E* ^−05^	0.000741	No match
gi|35090069	8.1	5.77*E* ^−05^	0.000741	*Saccharum* NCo 310 chloroplast DNA
gi|34977027	8.1	5.77*E* ^−05^	0.000741	*S. bicolor* mitochondrion
gi|35203085	8.1	5.77*E* ^−05^	0.000741	*S. bicolor *hypothetical protein
gi|35942526	8.1	5.77*E* ^−05^	0.000741	*S. bicolor *hypothetical protein
gi|35278085	8.1	5.77*E* ^−05^	0.000741	*S. bicolor* hypothetical protein
gi|35006270	8.1	5.77*E* ^−05^	0.000739	*Z. mays *GAPDH mRNA, partial cds
gi|35265314	8.1	5.77*E* ^−05^	0.000739	No match
gi|34957782	8.1	5.77*E* ^−05^	0.000739	*S. bicolor* hypothetical protein
gi|34964700	5.8	1.65*E* ^−05^	0.000243	*S. bicolor* hypothetical protein
gi|35245148	1.7	7.75*E* ^−07^	1.50*E* ^−05^	NSP-interacting kinase 1
gi|36032703	3.3	1.21*E* ^−08^	3.11*E* ^−07^	MKK6-putative MAPKK mRNA
gi|33461608	2.2	2.22*E* ^−06^	3.89*E* ^−05^	No match
gi|36001222	−16.2	0	0	Kladothrips maslini 16S ribosomal RNA
gi|35990824	−11.2	5.91*E* ^−37^	6.64*E* ^−35^	Manduca sexta actin mRNA, complete cds
gi|33461608	−10.3	2.74*E* ^−19^	2.03*E* ^−17^	No match
gi|36014330	−10.2	2.17*E* ^−18^	1.54*E* ^−16^	*Z. mays *subtilisin-chymotrypsin inhibitor,
gi|34922345	−9.5	1.67*E* ^−11^	6.06*E* ^−10^	*Z. perennis* isolate per7a MPI gene
gi|34971519	−9.3	2.63*E* ^−10^	8.38*E* ^−09^	*S. bicolor* hypothetical protein
gi|35975174	−8.5	4.09*E* ^−06^	6.81*E* ^−05^	*Z. mays *subtilisin-chymotrypsin inhibitor
gi|35056509	−8.3	1.63*E* ^−05^	0.000240497	*S. bicolor* hypothetical protein
gi|35008679	−8.1	6.45*E* ^−05^	0.000819694	*Z. mays *clone 261727 mRNA sequence
gi|35998230	−8.1	2.16*E* ^−85^	4.06*E* ^−83^	*Z. mays* subtilisin-chymotrypsin inhibitor
gi|36040747	−7.3	3.31*E* ^−48^	4.57*E* ^−46^	No match
gi|31072203	−6.5	6.96*E* ^−52^	1.02*E* ^−49^	No match
gi|35989329	−5.7	1.29*E* ^−58^	2.02*E* ^−56^	*Z. mays* adhesive/proline-rich protein
gi|35013208	−5.4	5.53*E* ^−37^	6.23*E* ^−35^	*S. bicolor* hypothetical protein
gi|36034301	−5.2	7.59*E* ^−14^	1.65*E* ^−141^	*Z. mays* xylanase inhibitor protein
gi|36008557	−5.1	1.67*E* ^−10^	5.41*E* ^−09^	*S. officinarum* clone SCQGLR1019F04
gi|34929144	−5.0	5.60*E* ^−52^	8.24*E* ^−50^	U-box domain-containing protein 21
