# Supplementary material for: Transcriptome Profile Analysis of Sugarcane Responses to Sporisorium scitaminea Infection Using Solexa Sequencing Technology
Source: Biomed Res Int. 2013 Oct 23;2013:298920. doi: 10.1155/2013/298920 (PMC3830884; doi:10.1155/2013/298920)

Statistics of Clean Tag Alignment

Mapping of Ya05-179-CK Total Clean Tags

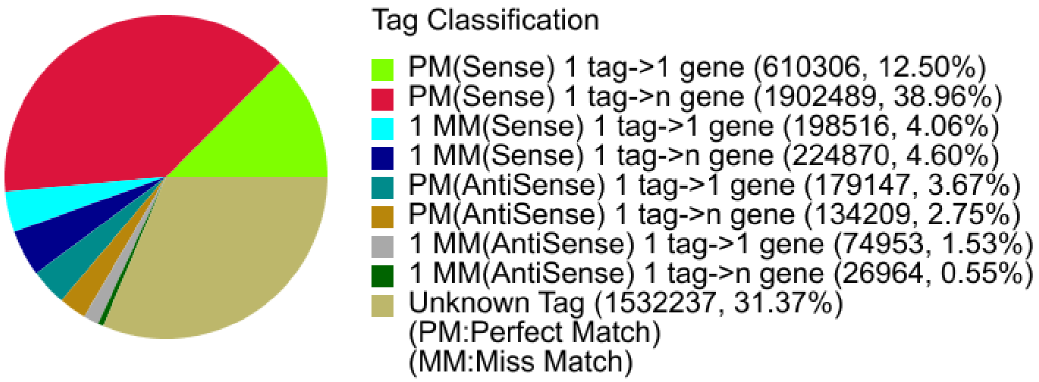

Mapping of Ya05-179-CK Distinct Clean Tags

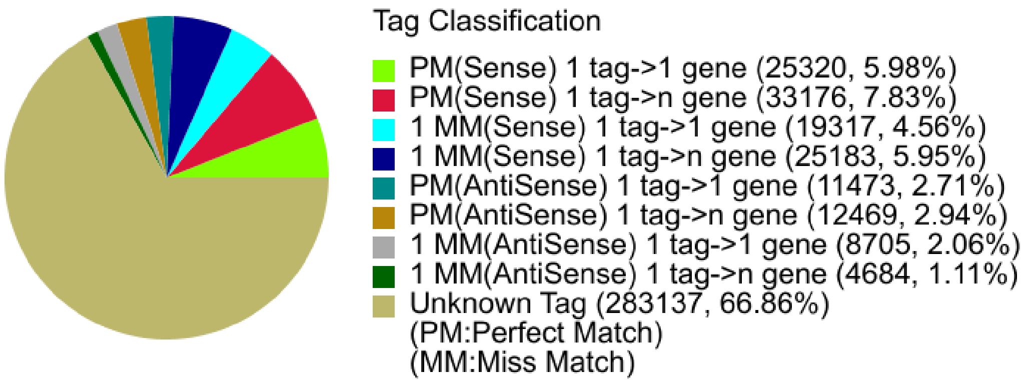

Mapping of Ya05-179-inoculation Total Clean Tags

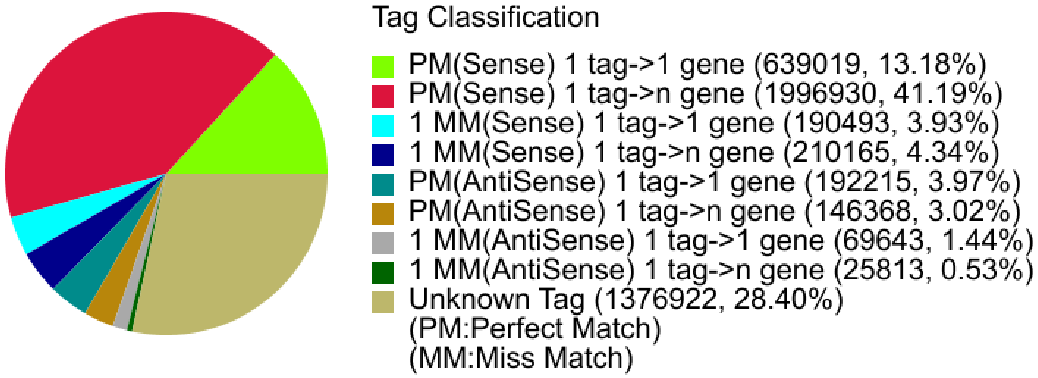

Mapping of Ya05-179-inoculation Distinct Clean Tags

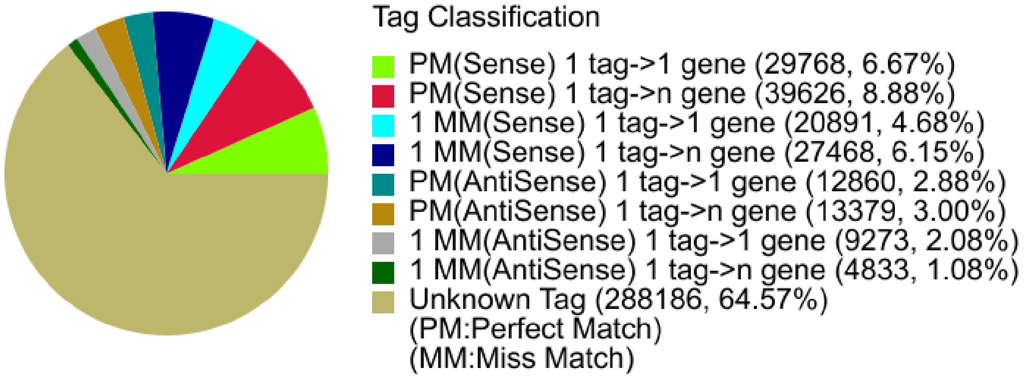

Supplement: Supplementary file 1 — Additional File 1: Statistics of clean tag alignment.pdf Additional File 2: Distribution of clean tag copy number.pdf Additional File 3: 2015 differentially expressed genes identified in sugarcane.xls Additional File 4: Gene ontology analysis for differentially expressed genes.xls Additional File 5: Pathway annotation of 305 differently expressed genes in sugarcane.xls Additional File 6: Raw data of qRT-PCR for ScBAK1, ScMapkk and ScGloI genes.xls [file 298920.f1.pdf]
