# Supplementary material for: Transcriptome Profile Analysis of Sugarcane Responses to Sporisorium scitaminea Infection Using Solexa Sequencing Technology
Source: Biomed Res Int. 2013 Oct 23;2013:298920. doi: 10.1155/2013/298920 (PMC3830884; doi:10.1155/2013/298920)

Distribution of Clean Tag Copy Number

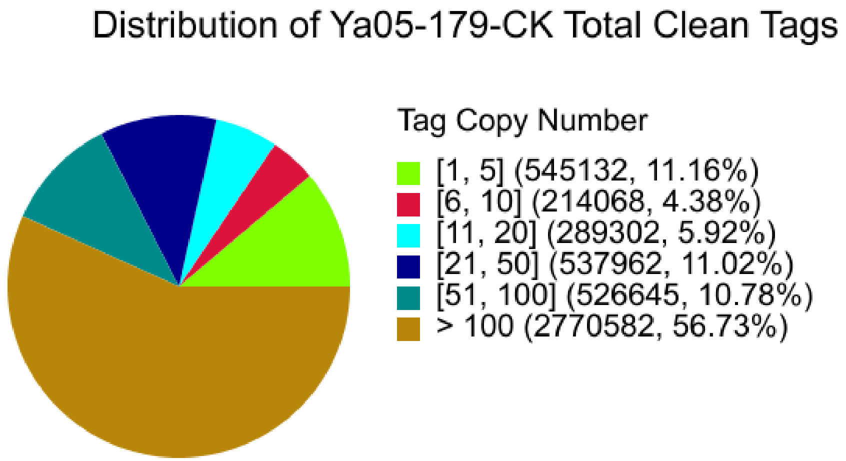

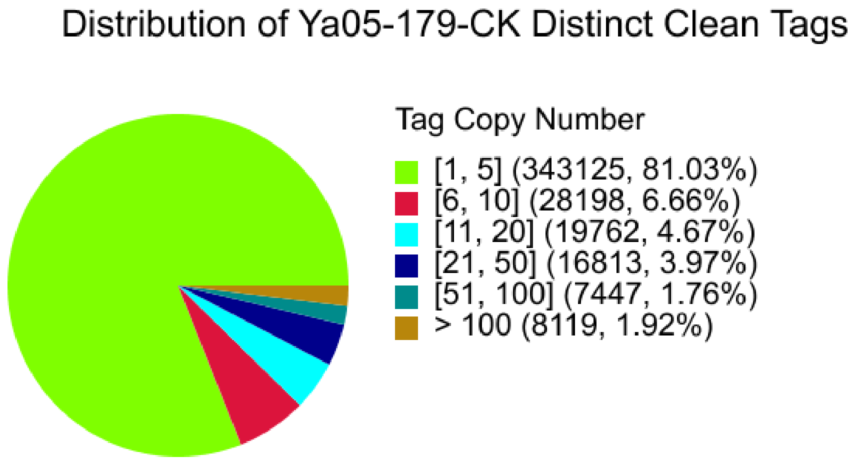

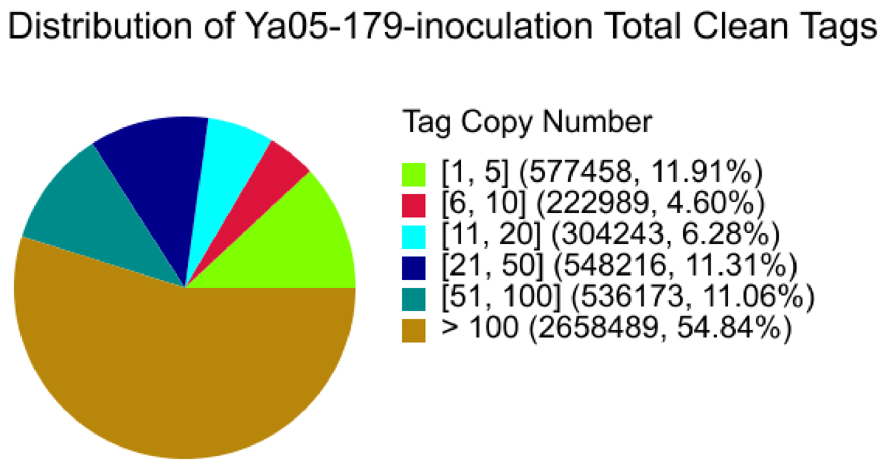

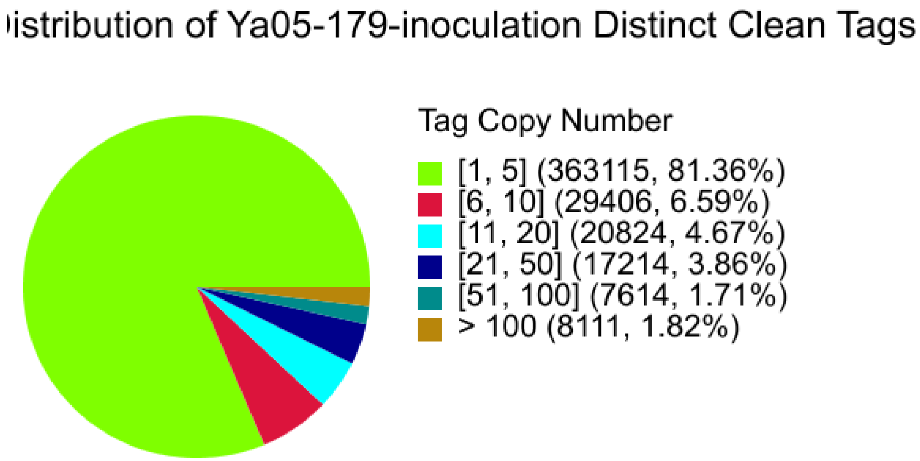

Supplement: Supplementary file 2 [file 298920.f2.pdf]
